# Genetic Variants of *SNCA* Are Associated with Susceptibility to Parkinson’s Disease but Not Amyotrophic Lateral Sclerosis or Multiple System Atrophy in a Chinese Population

**DOI:** 10.1371/journal.pone.0133776

**Published:** 2015-07-24

**Authors:** YongPing Chen, Qian-Qian Wei, RuWei Ou, Bei Cao, XuePing Chen, Bi Zhao, XiaoYan Guo, Yuan Yang, Ke Chen, Ying Wu, Wei Song, Hui-Fang Shang

**Affiliations:** 1 Department of Neurology and State Key Laboratory of Biotherapy and Cancer Center, West China Hospital, Sichuan University, Chengdu, Sichuan, China; 2 Department of Medical Genetics, West China Hospital, Sichuan University, Chengdu, Sichuan, China; Duke University, UNITED STATES

## Abstract

**Background:**

The polymorphisms of α-synuclein (*SNCA*), rs3775444, rs3822086 and rs11931074 that are strongly associated with Parkinson’s disease (PD) in Caucasian populations, were examined in this study to elucidate the role of polymorphisms in different ethnic backgrounds. The possible associations of these three polymorphisms were also investigated in PD, amyotrophic lateral sclerosis (ALS), and multiple system atrophy (MSA) in a Chinese population based on the overlapping of clinical manifestations and pathological characteristics of these three neurodegenerative diseases.

**Methods:**

A total of 1276 PD, 885 sporadic ALS (SALS), 364 MSA patients, and 846 healthy controls (HCs) were included. All subjects were genotyped for the three polymorphisms using Sequenom iPLEX Assay technology.

**Results:**

Significant differences in the genotype distributions (p = 5.99E-06 and p = 4.98E-06, respectively) and the minor allele frequency (MAF) (p = 2.16E-06 and p = 2.15E-06, respectively) of *SNCA* rs3822086 (C) and rs11931074 (G) were observed between PD and HCs. However, no differences were found in the genotype distributions and MAF of *SNCA* rs3775444 (T) between PD and HCs. Haplotype that incorporated the three SNPs further strengthened the association with PD (best haplotype, p = 9.62E-005). No significant differences in the genotype distributions and MAF of the SNPs were found between SALS and HCs, MSA and HCs, and subgroups of PD and SALS. However, the MAF of *SNCA* rs3775444 (T) was significantly higher in MSA patients with frontal lobe dysfunction than MSA patients without dysfunction (p = 0.0002, OR 2.53, 95%CI: 1.55-4.15).

**Conclusion:**

Our results suggest that the rs3822086 (C) allele and rs11931074 (G) allele in *SNCA* decrease the risk for PD, and *SNCA* rs11931074 may affect frontal lobe dysfunction of MSA in the Chinese population. However, these *SNCA* polymorphisms are not likely a common cause of SALS or MSA.

## Introduction

Parkinson’s disease (PD) is one of the most common age-related neurodegenerative disorders, and it affects approximately 1–2% of individuals older than 60 years. PD causes a progressive disability that may be slowed, but not halted, by treatment[1]. The cardinal pathological feature of PD is the selective loss of dopaminergic neurons in the substantia nigra pars compacta and the presence of Lewy bodies (LBs). However, the exact pathogenic mechanisms are not completely understood. Sporadic PD comprises approximately 90% of PD cases, but PD now has a well-established genetic component that includes disease-causing mutations and risk-modifying susceptibility variants[2]. However, the mutations that cause the monogenic form of PD only partially explain the familial aggregation of PD and rare sporadic cases. Many susceptibility variants were identified for PD. The best validated variants are those located in the α-synuclein (*SNCA*) gene, which plays an important role in the accumulation of the pathological hallmark of PD, LBs [3]. Mutations in the *SNCA* in PD patients are very rare. Whether susceptibility variants in the *SNCA* explain the abnormal intracellular protein aggregates in LBs is completely unknown. A previous study found that the rs356219 polymorphism in the 3′ region of the *SNCA* gene significantly affected on the expression of *SNCA* mRNA in human post-mortem brain tissue, including the substantia nigra and cerebellum [4]. A polymorphism, upstream of the *SNCA* also regulated α-synuclein expression in cultured SH-SY5Y cells [5]. Therefore, polymorphisms in the *SNCA* gene may importantly affect the risk of developing PD.

Ethnic specificity plays a crucial role in genetic heterogeneity. Recently, the minor alleles of polymorphisms rs2736990 and rs356220 in the *SNCA* were identified as a protective factor for PD in the Chinese population[6]. However, theses minor alleles increased PD risk for in the Caucasian population [7–9]. These inconsistent or contradictory results are caused by allelic heterogeneity because we noticed that the minor alleles of these two polymorphisms in Asian population are major alleles in Caucasian population. Therefore, other polymorphisms in the *SNCA* are worthy of investigation in non-Caucasian populations. The rs3822086 polymorphism in intron 4 of *SNCA* affects the phenotype of PD, including sex and the age of onset [8] and it is strongly associated with the cerebellar subtype of multiple system atrophy (MSA) [10]. The rs3775444 polymorphism in intron 4 of *SNCA* is associated with an increased risk for MSA, but not for PD [8, 10]. Another polymorphism, rs11931074, located downstream of the 3^’^ end of *SNCA*, is associated with PD and MSA in Caucasian populations [9, 11], but no association between the rs11931074 and MSA was found in the Chinese population[12].

Clinical phenotypes and pathological characteristics of PD, amyotrophic lateral sclerosis (ALS) and MSA also overlap. Epidemiological studies demonstrated that offspring of PD patients exhibit an increased risk for developing ALS [13], and ALS patients may develop associated features of Parkinsonism and movement disorders [14]. All of these diseases exhibit an aggregation of abnormal and misfolded proteins [15], such as TAR DNA-binding protein-43 (TDP43), SNCA, or tau, in cytoplasmic, nuclear and extracelluar inclusions, which suggests a common pathological pathway.

We performed a large case-control study on the associations between *SNCA* polymorphisms rs3775444, rs3822086 and rs11931074 and three neurodegenerative diseases (PD, ALS and MSA) in a Chinese population.

## Methods

### Subjects

Participants, including 1276 PD, 885 sporadic ALS (SALS), 364 MSA patients and 846 unrelated healthy controls (HCs), were recruited from the Department of Neurology, West China Hospital of Sichuan University. Clinical data of onset age, initial symptoms, family history, non-motor symptoms (including anxiety and depression), and frontal lobe function were recorded as described in previous studies in detail [6, 16]. Informed written consent was obtained from all participants prior to being recruitment. The Ethics Committee of West China Hospital of Sichuan University approved this study.

### Genotyping

Genomic DNA was extracted from peripheral blood leukocytes using standard phenol-chloroform procedures. The three selected SNPs, *SNCA* rs3775444, rs3822086 and rs11931074, were genotyped using Sequenom iPLEX Assay technology (Sequenom iPLEX Assay, San Diego) according to the manufacturer’s instructions. Approximately 10 ng of genomic DNA was used to genotype each sample. Locus-specific polymerase chain reaction (PCR) and detection primers were designed using Mass ARRAY Assay Design 3.0 software (Sequenom, San Diego). The DNA sample was amplified by primers flanking the targeted sequence, dephosphorylation and allele-specific primer extension. The PCR and extend primer sequences and conditions are shown in S1 Table. The extension products were purified and transferred to the 384-element SpectroCHIP bioarray. Parameters were obtained using Matrix-Assisted Laser Desorption/Ionization Time-of-Flight Mass Spectrometry (MALDI-TOF MS). The resultant data were processed and analyzed using Sequenom MassARRAY Workstation software (Sequenom).

### Statistical Analysis

Fisher’s exact test was performed to examine the Hardy–Weinberg equilibrium (HWE) for each locus in cases and controls separately. A Chi-squared test was used to compare differences in genotype frequencies between patients and controls. The role of each SNP was estimated by the minor allele frequency (MAF) and odds ratio (OR) with a 95% confidence interval (CI). All continuous data are presented as means ± standard deviation. Differences in continuous data, such as age of onset, between two groups were analyzed using Student’s t-test. A two-tailed *P*-value ≤0.05 was considered statistically significant, and p-values were corrected for multiple comparisons using the Bonferroni method (Pc = P/n, n was the number of tested SNPs). Three different genetic models, including dominant, recessive and additive were used for each SNP[17]. All analyses were performed using SPSS19.0 (SPSS, Inc., Chicago, IL, USA). The pairwise linkage disequilibrium parameter (D’) and haplotype associations were analyzed using SHEsis software[18]. Haplotypes comprised of five SNPs of *SNCA*, including rs3822086, rs11931074, rs3775444 and our previous published two SNPs[6], rs356220 and rs2736990, which are between rs11931074 and rs3775444, were analyzed in the PD group and healthy controls, which allowed further determinations of the *SNCA* gene region of risk/protection for PD in the Chinese population. The statistical power was calculated using PS Power and Sample Size Calculations software (version 3.0.43)[19].

## Results

### Clinical data and Genetic analysis

Table 1 shows the demographic information of the sample. A total of 484 PD patients, 312 SALS patients and 157 MSA patients were assessed for anxiety, depression and frontal lobe function.

**Table 1:** Clinical characteristic of patients and controls.

Variable	PD	SALS	MSA	HC
Cases, n	1276	885	364	846
Sex, female (%)	567 (45.14%)	356 (40.23%)	169 (46.43%)	370(43.74%)
Age (mean± SD)	60.45 ± 11.52	52.50 ± 11.35	59.53 ± 8.12	54.32 ± 12.78
Mean age of onset (mean ± SD)	56.32± 11.52	51.12 ± 12.09	56.54 ± 9.76	-
Mean disease duration (mean ± SD)	4.53 ± 4.15	1.84 ± 1.32	3.54 ± 7.59	-
Familial history	81 (6.35%)	-	-	-
FAB abnormal	45.04%(218/484)	39.10%(122/312)	60.51%(95/157)	
Anxiety	15.29%(74/484)	6.09%(19/312)	18.47%(29/157)	
Depression	17.77%(86/484)	6.73%(21/312)	12.10%(19/157)	

The genotype distributions of the three SNPs in all patients and control subjects did not significantly deviate from Hardy-Weinberg equilibrium (p > 0.05). Table 2 summarized the genotype and allele frequencies between patients and controls. Significant differences in genotype distributions and MAFs of rs3822086 (C) and rs11931074 (G) in *SNCA* were observed between PD and HCs. The dominant genetic model revealed that PD patients who carried the minor allele (“CC+CT” in the rs3822086 and “GG+GT” in the rs11931074, respectively) exhibited a decreased risk compared to patients who only carried the major allele (“TT” in the rs3822086 and “TT” in the rs11931074, OR 0.64, 95%CI: 0.53–0.77 and OR 0.63, 95%CI: 0.52–0.77, respectively). There were no differences in genotype distributions or MAFs of *SNCA* rs3775444 (T) between PD and HCs. No differences in genotype distributions and MAFs of all three SNPs were observed between SALS and HCs, or MSA and HCs.

**Table 2:** Genotyping Summary Statistics.

SNPs	Genotypes^※^					Genotype rate^☆^ (%)	HWE	MA	MAFs				
	PD	ALS	MSA	HCs	p-value^△^				PD	ALS	MSA	HCs	p-value^△^
rs3775444	173/575/516	117/398/365	53/160/151	105/386/348	0.74/0.88/0.59	99.29	0.90	T	36.43	35.91	36.54	35.52	0.55/0.81/0.63
rs3822086^∮^	208/598/465	203/422/258	87/163/113	181/432/226	*/0.31/0.11	99.56	0.34	C	39.89	46.89	46.42	47.32	^&^/0.80/0.69
rs11931074^∮^	205/600/464	205/420/256	86/162/113	178/437/225	^$^/0.20/0.07	99.41	0.20	G	39.80	47.11	46.26	47.20	^#^/0.95/0.67

HWE HCs: Hardy–Weinberg equilibrium of healthy controls; MAFs: minor allele frequencies; MA: minor allele

^※^genotypes for rs3775444 are TT, CT and CC, for rs3822086 are CC, CT and TT, for rs3822086 are GG, GT and TT, respectively

^△^ the p-value between PD and HCs, between ALS and HCs and between MSA and HCs from left to right, respectively

^☆^ success genotype rate for each SNP out of 3371 samples

*, p = 5.99E-06

^$^, p = 4.98E-06

^&^, p = 2.16E-06, OR 0.74[0.65, 0.84]

^#^, p = 2.15E-06, OR 0.74[0.65, 0.84]

^∮^PD: Dominant genetic model: rs3822086 CC+CT vs TT: OR 0.64, 95%CI 0.53–0.77, p = 4.155E-06

rs11931074GG+GT vs TT: OR 0.63, 95%CI 0.52–0.77, p = 2.617E-06

Haplotype analysis calculated the pairwise linkage disequilibrium (LD) coefficients (D^’^) between alleles of the *SNCA* SNPs (S2 Table). The five SNPs exhibited strong linkage disequilibrium between each pair, except a moderate linkage disequilibrium between rs3775444 and rs356220 and rs3775444 and rs2736990 was observed. The haplotypes “G-C-C” (34.8%) and “G-C-T” (1.4%) in PD patient comprised the minor alleles of rs11931074and rs3822086 (the first two alleles) decreased the risk for PD (OR 0.779, 95%CI: 0.687~0.883 and OR 0.479, 95%CI: 0.306~0.749, respectively), which suggests that the rs3775444 (the last allele did not affect the protective role of the haplotypes that were comprised of the minor alleles of rs11931074 (G) and rs3822086 (C) (shown in Table 3). Our findings were consistent with the individual effect of the minor alleles of rs11931074 and rs3822086 for PD. Notably, the haplotype “T-T-T” (35.1%) did not increase the risk for PD (OR 1.111, 95%CI: 0.975~1.267), but the “T-T-C” (25.0%) increased the risk for PD (OR 1.344, 95%CI: 1.157~1.563) (Table 3). Moreover, the haplotypes “G-C-C-C-C”, “G-C-C-T-C” and “G-C-C-T-T”, which incorporate 5 SNPs (rs11931074-rs356220-rs3822086-rs2736990-rs3775444 in order), also decreased the risk for PD. These results further determined the *SNCA* gene region of protection for PD in the Chinese population (Table 3).

Subgroups analysis revealed that the minor allele of *SNCA* rs3775444 increased the risk of frontal lobe dysfunction in MSA patients (p = 0.0002, OR 2.53, 95%CI: 1.55–4.15) (Table 4). However, no other significant differences in genotype distributions or MAF of the three SNPs were observed between subgroups regarding clinical presentations of PD, such as onset age, onset symptoms (tremor or rigidity), cognition (normal or abnormal), or anxiety and depression (presence or absence), clinical presentations of SALS, such as sex, onset age (EOALS and LOALS), and onset symptoms (spinal onset or bulbar onset), cognition (normal or abnormal), and anxiety or depression (presence or absence), and clinical presentations of MSA, such as sex, anxiety or depression (presence or absence) and subtypes of MSA (Table 4 and S3 Table).

**Table 3:** Comparison of SNPs haplotype in *SNCA* frequencies between patients and controls.

Haplotypes	Case (freq)	Control (freq)	Chi^2^	Fisher's-p	Pearson's-p	Odds Ratio [95%CI]
rs11931074-rs3822086-rs3775444-						
PD						
G-C-C	0.348	0.444	15.227	9.66e-005	9.62e-005	0.779 [0.687~0.883]
T-T-C	0.250	0.198	14.913	0.000114	0.000114	1.344 [1.157~1.563]
G-C-T	0.014	0.028	10.880	0.000979	0.000978	0.479 [0.306~0.749]
T-T-T	0.351	0.327	2.481	0.115318	0.115248	1.111 [0.975~1.267]
SALS						
G-C-C	0.444	0.444	0.000	0.985764	0.985756	1.001 [0.875~1.146]
T-T-C	0.194	0.198	0.089	0.765334	0.965325	0.975 [0.823~1.154]
G-C-T	0.025	0.028	0.308	0.579125	0.579123	0.888 [0.584~1.351]
T-T-T	0.334	0.327	0.178	0.673055	0.673056	1.031 [0.894~1.189]
MSA						
G-C-C	0.430	0.444	0.274	0.600966	0.600966	0.954 [0.799~1.138]
T-T-C	0.197	0.198	0.002	0.966049	0.966033	0.995 [0.799~1.240]
G-C-T	0.028	0.028	0.012	0.912565	0.912539	1.030 [0.608~1.745]
T-T-T	0.337	0.327	0.302	0.582880	0.582878	1.503 [0.875~1.268]
rs11931074-rs356220*-rs3822086-rs2736990*-rs3775444						
PD						
G-C-C-C-C	0.045	0.077	15.96	6.56e-005	6.53e-005	0.560[0.420–0.747]
G-C-C-T-C	0.300	0.349	9.150	0.002500	0.002497	0.799[0.691–0.924]
G-C-C-T-T	0.010	0.022	7.269	0.007037	0.007030	0.473[0.271–0.825]
G-T-C-T-C	0.015	0.017	0.136	0.712662	0.712660	0.904[0.528–1.547]
T-T-T-C-C	0.223	0.193	4.598	0.032050	0.032023	1.203[1.016–1.424]
T-T-T-C-T	0.315	0.318	0.027	0.869146	0.869123	0.988[0.853–1.144]

*The genotype data come from our previous study

**Table 4:** Association between two SNPs and frontal lobe function, anxiety and depression of patients in PD, SALS and MSA patients.

	rs3775444				rs3822086				rs11931074			
	TT/CT/CC	*P* value	MAF (%)	*P* value	CC/CT/TT	*P* value	MAF (%)	*P* value	GG/GT/TT	*P* value	MAF (%)	*P* value
PD												
FAB												
abnormal (score<16)	19/114/82	0.04	35.35	0.80	36/114/69	0.44	42.47	0.44	36/115/68	0.36	42.69	0.35
normal (score ≥16)	39/115/113		36.14		45/126/99		40.00		44/125/99		39.74	
Anxiety												
presence (score>14)	11/33/28	0.64	38.19	0.54	12/36/24	0.97	41.67	0.86	12/36/24	0.98	41.67	0.85
absence (score≤14)	46/196/163		35.56		68/201/143		40.90		67/201/142		40.85	
Depression												
presence (score>20)	8/39/37	0.56	32.74	0.29	15/38/31	0.70	40.48	0.93	15/38/31	0.70	40.48	0.93
absence (score≤20)	50/194/153		37.03		63/202/137		40.84		63/202/137		40.80	
SALS												
FAB												
abnormal (score<16)	16/68/68	0.82	32.89	0.83	38/69/44	0.66	48.01	0.73	38/70/43	0.77	48.34	0.80
normal (score ≥16)	27/92/98		33.64		53/110/56		49.32		53/109/56		49.31	
Anxiety												
presence (score>14)	1/6/11	0.35	22.22	0.14	7/5/6	0.24	52.78	0.73	7/5/6	0.25	52.78	0.73
absence (score≤14)	38/123/130		34.19		76/140/77		49.83		76/139/77		49.83	
Depression												
presence (score>20)	3/6/11	0.55	30.00	0.63	6/5/9	0.09	42.50	0.33	6/5/9	0.09	42.50	0.33
absence (score≤20)	36/123/130		33.74		77/140/74		50.52		77/139/74		50.52	
MSA												
FAB												
abnormal (score<16)	23/45/28	0.001	47.40	0.0002*	22/38/36	0.15	42.71	0.05	22/38/36	0.22	42.71	0.05
normal (score ≥16)	7/18/36		26.23		19/28/14		54.10		19/27/14		54.17	
Anxiety												
presence (score>14)	5/13/12	0.99	38.33	0.96	6/16/8	0.63	46.67	0.98	6/16/8	0.63	46.67	0.97
absence (score≤14)	22/55/51		38.67		32/56/40		46.88		31/55/40		46.43	
Depression												
presence (score>20)	1/12/6	0.12	36.84	0.81	3/11/5	0.48	44.74	0.78	3/11/5	0.49	44.74	0.82
absence (score≤20)	26/56/57		38.85		35/61/43		47.12		34/60/43		46.72	

FAB: frontal assessment battery

*OR 2.53, 95%CI: 1.55–4.15

## Discussion

This large-scale study investigated the genetic susceptibility to neurodegenerative diseases and analyzed associations between *SNCA* alleles and PD, SALS, and MSA. The present study demonstrated that the *SNCA* rs3822086 and rs11931074 decreased the risk for PD, and the *SNCA* rs3775444 increased the risk for developing frontal lobe dysfunction of MSA. This exploratory study is the first investigation of the association between these three candidate polymorphisms and SALS. However, these three polymorphisms were not the risk factors for SALS.

The *SNCA* gene undoubtedly plays an important role in PD because mutations in *SNCA* cause PD and variants in *SNCA* affect the susceptibility to PD. However, PD patients who carry mutations in *SNCA* are rare (http://www.hgmd.cf.ac.uk/ac/all.php). α-synuclein is a primary protein component of LBs, and it is involved in the common mechanisms of α-synuclein and tau aggregation [20]. Variants in *SNCA* regulate changes in α-synuclein levels and alternative transcripts intracellularly, which may account for the association with the risk for PD [4, 5].

Ethnicity plays an important role in genetic heterogeneity. We recently found that the minor alleles of *SNCA* rs2736990 and rs356220 were protective factors for PD in the Chinese population[6]. However, these minor alleles are risk factors for PD in Caucasians [7]. This discrepancy prompted us to investigate the role of other SNPs in this gene in PD risk. The current study demonstrated that the rs3822086 (C) allele decreased the risk for PD, but this only affected the sex and onset age of PD in Caucasians [8]. The rs11931074 (G) allele decreased the risk for PD in our study, which is consistent with the findings from other Asian studies, including a Korean study [21] and some Chinese studies [22–24]. However, the rs11931074 (T) allele increased the risk for PD in Caucasians because of the allelic heterogeneity between different ethnics [9]. Notably, the rs3822086 and rs11931074 alleles exhibited nearly complete linkage disequilibrium, which further supports the results that the rs3822086 allele decreased the risk for PD in Chinese population. The rs3775444 allele did not increase the risk for PD or affect the clinical phenotype of PD in Chinese population, which is similar to a Caucasian study [8]. However, the haplotype “T-T-C” comprised by rs11931074, rs3822086 and rs3775444 increased the risk for PD, but “T-T-T” did not, which suggests that the rs3775444 (T) allele is a protective factor in the haplotype model. The hypothesis requires confirmation in future studies. The decreased risk in three haplotypes primarily comprised by the minor alleles of five *SNCA* SNPs further localized the *SNCA* gene region of protection for PD. Nevertheless, few studies investigated the possible mechanism of SNPs in PD, including our study, which primarily focused on the association between α-synuclein expressions at the transcript and protein levels. However, the results are conflicting. For example, one study found that the rs11931074 (T) allele was associated with decreased α-synuclein in serum [21], but, another study did not find any association between the rs11931074 (T) allele and transcript isoform expression of SNCA in the brain [22]. Therefore, more functional studies are needed to elucidate the possible mechanism of SNPs in PD. Moreover, subgroups analysis revealed that the SNPs in the *SNCA* did not contribute to the clinical phenotype of PD, such as sex, onset symptoms, cognitive dysfunction, anxiety or depression.

The rs3775444 and rs3822086 alleles in the *SNCA* did not increase the risk for developing MSA or MSA-C whatever in a dominant, recessive or additive model in this study (data no shown), which is different from the study on Caucasians [10] The current study exhibited a 95.6% power to detect an odds ratio of 1.889 for an MAF of 5.22% in controls for the rs3822086, and a 96.6% power to detect an odds ratio of 2.443 for an MAF of 2.41% in controls for the rs3775444 [10]. However, MSA patients with the rs3775444 (T) allele were susceptible to frontal lobe dysfunction. The underlying mechanism is not known. Further studies on the associations between *SNCA* rs3775444 and cognition-related diseases, such as Alzheimer’s disease and frontotemporal dementia (FTD), will help confirm this association. A previous GWAS study demonstrated that the *SNCA* rs11931074 exhibited the strongest association with an increased risk for MSA only in a recessive genetic model, but no difference or a weak correlation was observed in the allelic, dominant, and trend models in the screening stage, the replication stage, or the combined analysis [11]. These results suggest that *SNCA* rs11931074 and rs3822086 play a limited role in MSA in the Chinese population [10–12].

This exploratory study did not identify no association between the candidate *SNCA* SNPs and SALS, which is consistent with our previous study on the association between other *SNCA* variants and SALS [6]. These results suggest a limited role of the *SNCA* gene in the development of ALS. The SNPs in the *SNCA* also did not contribute to other clinical features of ALS, such as sex, onset age, onset forms, cognitive dysfunction, anxiety or depression.

In conclusion, our results strongly support the susceptibility function of *SNCA* polymorphisms in the Chinese population and provide evidence that the rs3822086 (C) allele and rs11931074 (G) allele in *SNCA* decrease the risk for PD. The SNP rs11931074 in *SNCA* may affect frontal lobe dysfunction of MSA in the Chinese population. The three candidate SNPs in *SNCA* are not likely the causes of ALS and MSA.

## Supporting Information

S1 Table PCR and extend primers and conditions. (DOCX)

S2 Table Linkage disequilibrium tests for SNPs in SNCA. (DOCX)

S3 Table Analysis of the genotype distribution of three SNPs in patients. (DOCX)
