# Supplementary material for: Genetic Variants of SNCA Are Associated with Susceptibility to Parkinson’s Disease but Not Amyotrophic Lateral Sclerosis or Multiple System Atrophy in a Chinese Population
Source: PLoS One. 2015 Jul 24;10(7):e0133776. doi: 10.1371/journal.pone.0133776 (PMC4514852; doi:10.1371/journal.pone.0133776)
Supplement: S1 Table — (DOCX) [file pone.0133776.s001.docx]

S1 Table. PCR and extend primers and conditions

|  | rs3775444 | rs3822086 | rs11931074 |
| --- | --- | --- | --- |
| PCR- Forward primer | ACGTTGGATGTTGGTACTGAAGGTCACAAG | ACGTTGGATGCAGAGGTGTTTTGCCTAAAG | ACGTTGGATGTCTTCCTCGGAAGAGATACC |
| PCR- Reverse primer | ACGTTGGATGACTCTGTGCTCACAGTGTTC | ACGTTGGATGCATGCTTTGACGTAAGACTG | ACGTTGGATGACAGTCAAATGGCAGCCTTC |
| PCR conditions | 94℃,15min；94℃,20sec,56℃,30sec72℃,1min(45cycles);72℃,3min; | | |
| Extend primer-1 | ACAAACAAAAAACAACCAC | AACCCAGCTTGACTATC | GGATCCTTCCAAATCATAATTCCCT |
| Extend primer-2 | ACAAACAAAAAACAACCACC | AACCCAGCTTGACTATCA | GGATCCTTCCAAATCATAATTCCCTC |
| Extend primer-3 | ACAAACAAAAAACAACCACT | AACCCAGCTTGACTATCG | GGATCCTTCCAAATCATAATTCCCTA |
| Extend conditions | 94℃,30sec;94℃,5sec,52℃,5sec(40cycles);94℃,5sec,80℃,5sec(5cycles);72℃,3min; | | |
