# Supplementary material for: Genetic Variants of SNCA Are Associated with Susceptibility to Parkinson’s Disease but Not Amyotrophic Lateral Sclerosis or Multiple System Atrophy in a Chinese Population
Source: PLoS One. 2015 Jul 24;10(7):e0133776. doi: 10.1371/journal.pone.0133776 (PMC4514852; doi:10.1371/journal.pone.0133776)
Supplement: S2 Table — (DOCX) [file pone.0133776.s002.docx]

S2 Table. Linkage disequilibrium tests for SNPs in *SNCA*

| D^’^ | rs356220 | rs3822086 | rs2736990 | rs3775444 |
| --- | --- | --- | --- | --- |
| rs11931074 | 0.878 | 0.995 | 0.917 | 0.884 |
| rs356220 | - | 0.878 | 0.910 | 0.678 |
| rs3822086 | - | - | 0.921 | 0.881 |
| rs2736990 | - | - | - | 0.780 |
