# Supplementary material for: Genetic Variants of SNCA Are Associated with Susceptibility to Parkinson’s Disease but Not Amyotrophic Lateral Sclerosis or Multiple System Atrophy in a Chinese Population
Source: PLoS One. 2015 Jul 24;10(7):e0133776. doi: 10.1371/journal.pone.0133776 (PMC4514852; doi:10.1371/journal.pone.0133776)
Supplement: S3 Table — (DOCX) [file pone.0133776.s003.docx]

S3 Table. Analysis of the genotype and minor allele frequencies distribution of three SNPs in patients

|  | rs3775444 | | | |  | rs3822086 | | | | |  | rs11931074 | | | | |
| --- | --- | --- | --- | --- | --- | --- | --- | --- | --- | --- | --- | --- | --- | --- | --- | --- |
|  | TT/CT/CC | *P* value | MAF (%) | *P* value |  | CC/CT/TT | *P* value | | MAF (%) | *P* value |  | GG/GT/TT | *P* value | | MAF (%) | *P* value |
| PD |  | |  |  |  |  | | |  |  |  |  | | |  |  |
| sex |  | |  |  |  |  | | |  |  |  |  | | |  |  |
| males | 95//318/278 | 0.89 | 36.76 | 0.71 |  | 104/342/253 | | 0.19 | 39.34 | 0.53 |  | 103/343/252 | | 0.22 | 39.33 | 0.48 |
| females | 78/257/238 |  | 36.04 |  |  | 104/256/212 | |  | 40.56 |  |  | 102/257/212 | |  | 40.72 |  |
| Onset age |  |  |  |  |  |  | |  |  |  |  |  | |  |  |  |
| <50ys | 48/156/125 | 0.51 | 38.30 | 0.25 |  | 49/148/133 | | 0.25 | 37.27 | 0.11 |  | 49/146/134 | | 0.19 | 37.08 | 0.10 |
| ≥50ys | 125/419/391 |  | 35.78 |  |  | 159/450/332 | |  | 40.81 |  |  | 156/454/330 | |  | 40.74 |  |
| Initial symptoms |  |  |  |  |  |  | |  |  |  |  |  | |  |  |  |
| Tremor | 74/201/201 | 0.003^*^ | 36.66 | 0.53 |  | 87/205/182 | | 0.092 | 39.98 | 0.14 |  | 87/206/181 | | 0.10 | 40.08 | 0.14 |
| Rigidity | 51/196/163 |  | 36.34 |  |  | 63/203/150 | |  | 39.54 |  |  | 62/202/150 | |  | 39.37 |  |
| Mixed symptoms | 6/43/45 |  | 34.69 |  |  | 13/52/33 | |  | 39.80 |  |  | 13/52/33 | |  | 39.80 |  |
| Bradykinesia | 3/17/36 |  | 42.62 |  |  | 4/29/30 | |  | 29.37 |  |  | 4/29/30 | |  | 29.37 |  |
| ALS |  |  |  |  |  |  | |  |  |  |  |  | |  |  |  |
| sex |  |  |  |  |  |  | |  |  |  |  |  | |  |  |  |
| males | 69/243/213 | 0.71 | 36.29 | 0.68 |  | 117/249/162 | | 0.44 | 45.74 | 0.22 |  | 118/249/160 | | 0.50 | 46.02 | 0.24 |
| females | 48/154/152 |  | 35.31 |  |  | 86/173/95 | |  | 48.73 |  |  | 87/171/95 | |  | 48.87 |  |
| Onset age |  |  |  |  |  |  | |  |  |  |  |  | |  |  |  |
| ＜40ys | 20/79/69 | 0.74 | 35.42 | 0.83 |  | 41/82/45 | | 0.72 | 48.81 | 0.43 |  | 41/82/45 | | 0.76 | 48.81 | 0.49 |
| ≥40ys | 97/319/296 |  | 36.03 |  |  | 162/340/213 | |  | 46.43 |  |  | 164/338/211 | |  | 46.79 |  |
| Initial symptoms |  |  |  |  |  |  | |  |  |  |  |  | |  |  |  |
| Limbs | 82/307/282 | 0.28 | 35.10 | 0.65 |  | 153/325/195 | | 0.75 | 46.88 | 0.56 |  | 153/324/194/ | | 0.58 | 46.94 | 0.40 |
| Spinal | 29/73/78 |  | 36.39 |  |  | 46/84/51 | |  | 48.62 |  |  | 48/83/50 | |  | 49.45 |  |
| MSA |  |  |  |  |  |  | |  |  |  |  |  | |  |  |  |
| sex |  |  |  |  |  |  | |  |  |  |  |  | |  |  |  |
| males | 27/83/83 | 0.82 | 35.49 | 0.53 |  | 50/87/56 | | 0.54 | 48.45 | 0.24 |  | 49/85/57 | | 0.65 | 47.91 | 0.35 |
| females | 26/77/68 |  | 37.72 |  |  | 37/76/57 | |  | 44.12 |  |  | 37/77/56 | |  | 44.41 |  |
| Phenotype |  |  |  |  |  |  | |  |  |  |  |  | |  |  |  |
| MSA-C | 30/77/68/ | 0.40 | 39.14 | 0.22 |  | 44/69/62 | | 0.24 | 44.86 | 0.27 |  | 44/68/62 | | 0.22 | 44.83 | 0.32 |
| MSA-P | 13/51/49 |  | 34.07 |  |  | 29/54/30 | |  | 49.56 |  |  | 28/54/30 | |  | 49.11 |  |

*no difference after adjustment
